# The LOX-1 receptor ectopically expressed in the liver alleviates atherosclerosis by clearing Ox-LDL from the circulation

**DOI:** 10.1186/s10020-022-00450-3

**Published:** 2022-03-02

**Authors:** Zhiwen Wang, Juan Chen, Zhuanglin Zeng, Qing Zhang, Gaohui Du, Xiaopeng Guo, Yumiao Wei

**Affiliations:** 1 Department of Cardiology, Union Hospital, Tongji Medical College, Huazhong University of Science and Technology, Wuhan, China; 2 Department of Emergency Medicine, Union Hospital, Tongji Medical College, Huazhong University of Science and Technology, Wuhan, China; 3 Department of Radiology, Union Hospital, Tongji Medical College, Huazhong University of Science and Technology, Wuhan, China

**Keywords:** Ox-LDL, LOX-1, Liver, AAV8, Atherosclerosis

## Abstract

**Objective:**

Oxidized Low-Density-Lipoprotein (Ox-LDL) is the core factor in the development of atherosclerosis. However, there are few therapies aimed at eliminating Ox-LDL. Here in this study, we investigate whether the ectopically expression of the lectin-like oxidized low density lipoprotein receptor (LOX-1) in the liver could lead to the elimination of circulating Ox-LDL and prevent the deposition in the vascular wall, thereby alleviating the progression of atherosclerosis.

**Methods:**

Apolipoprotein E-deficient (ApoE^−/−^) mice were randomly divided into three groups, the control group, the AAV8-TBG-eGFP group (eGFP group) and AAV8-TBG-LOX-1 group (LOX-1 group). In the LOX-1 group, mice received an injection of virus dilution AAV8-TBG-LOX-1 (1.16 × 10^11^ virus genome (v.g)/animal/100 μl). The mice in the control group and eGFP group received the same amount of sterile saline and AAV8-TBG-eGFP virus dilution injections. The expression of LOX-1 in the liver was detected by immunofluorescent, western blot and immunohistochemistry. The safety of the virus was assessed by hematoxylin–eosin (H&E) staining, blood biochemical analyses and immunofluorescent. The function of LOX-1 in the liver was detected by the co-localization of LOX-1 and Dil-labeled Ox-LDL (Dil-Ox-LDL) under laser scanning confocal microscope. The extent of Ox-LDL in plasma was detected by ELISA. Changes in blood lipids were assessed through blood biochemical analysis. The progression of atherosclerotic lesions was detected by Oil red O staining. And the expression of Vascular Cell Adhesion Molecule-1 (VCAM-1) in endothelial cells and the extent and migration of macrophages in atherosclerotic plaque were detected by immunofluorescence staining. The protein expression in liver was assessed by qRT-PCR and western blot.

**Results:**

The expression of LOX-1 was stable in liver within 4 weeks. Ectopically expressed LOX-1 in the liver phagocytosed and degraded Ox-LDL and reduced Ox-LDL from circulation but did not have a significant effect on blood lipid levels. After the expression of LOX-1 in liver, Ox-LDL can be cleared by the hepatocytes, thereby reducing VCAM-1 expression in vascular endothelium and the migration of macrophages in plaques, and eventually alleviating the progression of atherosclerosis. Functional expression of LOX-1 in hepatocytes may facilitate the metabolic clearance of Ox-LDL by upregulating the expression of ATP-binding cassette G5 and G8 (ABCG5/G8), which is the primary neutral sterol transporter in hepatobiliary and transintestinal cholesterol excretion.

**Conclusion:**

Ectopic liver-specific expression of LOX-1 receptor alleviates the progression of atherosclerosis by clearing Ox-LDL from circulation.

## Introduction

Atherosclerosis is a systematic pathological change caused by the abnormal deposition of cholesterol-rich lipoproteins in the vascular wall (Libby et al. 2019). Atherosclerotic cardiovascular diseases account for the leading cause of death and the majority of the world’s disease burden, of which the incidence has continued to increase by 21.1% in the last decade (Collaborators GBDCoD 2018; Julius 2017). Based on the previous research and practice on atherosclerosis, modern clinical practice can lower low-density lipoprotein (LDL) cholesterol to a very low state with drugs such as statins to inhibite the progression of atherosclerosis (Dullaart 2017). However, as the world's leading fatal disease, the complexity of its pathogenesis is still far from being completely understood, and simply lowing LDL is not enough. Atherosclerotic interventions still need to be explored from multiple perspectives to develop effective treatment methods.

Although lipid metabolism disorders are the leading cause of atherosclerosis, the deposition of Ox-LDL and lipid-driven inflammatory responses in the vascular wall are the primary mechanism of atherosclerotic formation (Libby and Hansson 2019; Libby 2002; Grootaert and Bennett 2021). Ox-LDL in atherosclerotic patients not only exists in atherosclerotic plaques deposited in the vascular wall but also exists in circulating plasma. There are more than 20 clinical studies on the association between Ox-LDL and atherosclerotic cardiovascular disease, and several studies enrolled 1000 to 2000 atherosclerosis patients. According to the study of Gao et al., after correcting for the exclusion of LDL cholesterol risk factors, the risk of atherosclerotic cardiovascular disease in patients with elevated Ox-LDL increased 1.66–2.88 times (Gao et al. 2017). Several case–control studies were divided into four components based on Ox-LDL levels. Excluding the influencing factors of LDL cholesterol, the risk of cardiovascular disease in 14 groups with the highest Ox-LDL level and 14 groups with the lowest Ox-LDL level increased 1.67–5.03 times, with an average increase in cardiovascular events of approximately 2.93 times for every 1 U increase in circulating Ox-LDL (Gao et al. 2017). Ox-LDL levels, including circulating Ox-LDL levels, are a strong risk factor independent of LDL cholesterol. According to the reports of Sabatine et al. and Schwartz et al., patients who used PCSK9i, the level of LDL in the circulation reduced to approximately 30 mg/dl (0.78 mmol/l), which is near the physiological limit of cholesterol in the human body. As the results, after 3 and 4 years of follow-up, the rates of cardiovascular events in these patients from 14.6% in the control group to 12.6% in the treatment group and from 11.1% in the control group to 9.5% in the treated group, respectively (Szarek et al. 2019; Sabatine et al. 2017). So, we can see that, although the level of LDL almost has been reduced to the physiological limit, there is still a limited reduction in atherosclerotic cardiovascular events. Hence, lowering plasma LDL alone could significantly reduce Ox-LDL levels but could not completely inhibit Ox-LDL production and macrophage phagocytosis as well as vascular wall deposition. Fundamentally reducing the deposition of Ox-LDL in the vascular wall should be a more specific treatment strategy.

Existing drugs do not sufficiently inhibit the oxidation of circulating and tissue lipids because excess LDL is too easy to oxidize and modify. The ideal way to prevent the progression of atherosclerosis is for the LDL level to be as low as possible, resulting in the lowest possible initiation of lipid oxidation and inflammation. The proposed approach is to convert the clearance mechanism for circulating pathogenic lipid particles from inflammatory clearance mechanisms by the existing innate immune system to normal metabolic mechanisms. In this field, the existing lipid-lowering drugs have achieved sufficient LDL-lowering effects, but the clearance of Ox-LDL remains a problem (Mortensen and Nordestgaard 2020; Bonaca and Hess 2020). Theoretically, the adequate clearance of LDL to avoid oxidation and facilitate its removal can inhibit the deposition of atherosclerotic lipids and inflammation processes. Therefore, the clearance of Ox-LDL may be more meaningful. Among the possible strategies, the aim of this study was to start with the LOX-1 scavenger receptor and liver lipid metabolism to explore possible ways to achieve this goal.

LOX-1 is a transmembrane glycoprotein that, structurally, is in the C-type lectin family and a class ESR (a scavenger receptor with a haemagglutinin structural domain). It is mainly expressed in vascular endothelial cells but can also be expressed in macrophages, dendritic cells and fibroblasts. The expression level of the LOX-1 receptor in vascular endothelial cells is very low under physiological conditions, but it is significantly upregulated during oxidative stress and inflammatory responses (Akhmedov et al. 2021). Ox-LDL is the major specific ligand of the LOX-1 receptor. Vascular endothelial cells mainly bind, phagocytose and degrade Ox-LDL through the LOX-1 receptor, thereby causing endothelial cell dysfunction and associated damage (Kattoor et al. 2019). For the scavenger receptors mentioned above, as a transmembrane glycoprotein receptor that binds and internalizes Ox-LDL, LOX-1 differs from CD36 and SR-A receptors: the ligands recognized by the latter two are more extensive and can only conduct phagocytosis after the extensive oxidation of LDL particles, while LOX-1 is more specific for Ox-LDL and can also interact with mildly oxidated LDL. Therefore, LOX-1 may have better prospects as a potential intervention target for the clearance of Ox-LDL (Yoshimoto et al. 2011; Negre-Salvayre et al. 2017).

As the hub of metabolic transformation of the entire body, the liver has a highly sophisticated metabolic system and is also the core organ for lipid metabolism and transformation. Unlike macrophages and endothelial cell pattern recognition receptors, which have no downregulation function after Ox-LDL phagocytosis, hepatocyte lipid metabolism receptors are involved in downregulation functions and multiple metabolic regulatory pathways. A large number of hepatocytes, lipid metabolism factories, cannot exert their important metabolic clearance function to Ox-LDL simply because they lack corresponding receptors. With the help of existing highly specific liver transgenic expression technology (Lehrke and Lebherz 2014; Colella et al. 2018; Hacker et al. 2020), this study envisioned the use of adeno-associated virus type 8 (AAV8) containing a liver-specific thyroxine binding globulin (TBG) promoter as a vector to mediate the ectopic expression of LOX-1receptor in hepatocytes to clear Ox-LDL from the circulation (Geng et al. 2017; Wang et al. 2012). We hope to achieve the physiological degradation of Ox-LDL so as to avoid lipid deposition in the vascular wall and atherosclerosis formation through multiple lipid metabolism pathways in hepatocytes, especially Ox-LDL lysosomal degradation and biliary cholesterol excretion, which are two major hepatic pathways for handling excess lipid molecules.

## Materials and methods

### Study design and animals

AAV expressing LOX-1 fused with enhanced green fluorescent protein (eGFP) under the control of hTBG promotor, the vector backbone (plasmid) is WY3081 (Shanghai Genechem Co., Ltd). The high titers of engineered AAV (AAV2/8-hTBG-LOX-1-P2A-eGFP-3xFlag-WPRE-SV40pA, 1.16 × 10^13^ virus genome (v.g)/ml) and the negative control virus (AAV2/8-hTBG-eGFP-WPRE-pA, 1.76 × 10^13^ v.g/ml) were produced by Shanghai Genechem Co., Ltd. In order to test the specific transfection effect of the AAV in vivo, we use the negative control virus (1.76 × 10^13^ v.g/animal/100 μl) for pre-experiment.

Male ApoE^−/−^ mice were obtained at 6 weeks of age from Hua Fukang Experimental Animal Center (Beijing, China). Mice were selected and housed in a single-cage single-chamber system at the specific-pathogen-free (SPF) grade Animal Experimental Center, Tongji Medical College, Huazhong University of Science and Technology. They were fed a high-fat diet consisting of 1.25% cholesterol and 20% fat (Special Diet Services, D12079B) for 8 weeks to induce atherosclerosis. Body weight was measured weekly and recorded. The condition of the mice was observed. Mice were randomly divided into three groups: the control group, the eGFP group and the LOX-1 group. The first intervention was conducted in the first week (7 weeks old), and the second intervention was performed in the 5th week. Mice were sacrificed the 1st week, 2nd week, 3rd week, 4th week and 8th week after virus injection, and samples were collected for the following analyses.

Intervention programme: AAV8-TBG-LOX-1 vector (1.16 × 10^13^ v.g/animal/100 μl) and AAV8-TBG-eGFP vector (1.76 × 10^13^ v.g/animal/100 μl) was freshly prepared by diluting the vector to a viral titre of 1.16 × 10^11^ v.g/ml (the titre of AAV8-TBG-eGFP is 1.76 × 10^11^ v.g/ml) with sterile phosphate-buffered saline (PBS) and then stored in an ice box to avoid the influence of repeated freeze–thaw cycles on the virus titre. For the LOX-1 group and the eGFP group, 100 μl virus dilution was injected through tail vein, and for the control group, 100 μl of sterile saline was injected through the tail vein.

### Blood collection

To avoid the influence of Dil-labelled Ox-LDL (Dil-Ox-LDL) (Yiyuan Biotech, YB-0010) on the Ox-LDL content in the circulation, we collected blood from the orbital intracanthal vein before the probe injection. Mice were fasted for 8 h before blood collection, and the glass capillary tube was tilted at 30° and slowly rotated to puncture the angular vein for blood collection. After the blood sample is collected, use the corresponding ELISA (CUSABIO, CSB-E07933m) kit to detect Ox-LDL in serum. The specific operation steps are as described in the previous study (Zeng et al. 2018). Blood collection from the orbital sinus by quickly removing the eyeball from the socket (the mice were sacrificed) was used for the detection of other blood parameters, such as ALT, AST, etc. The blood sample obtained from the above method was placed at room temperature for three hours and then centrifuged at room temperature for 15 min at 3500 r/min. The supernatant was collected in an Eppendorf (EP) tube and stored in a − 80 °C freezer.

### Blood lipid panel and biochemical indices

A fully automated biochemical analyser was used to detect the levels of blood lipids, namely, total cholesterol (TC), triglyceride (TG), high-density lipoprotein (HDL), and Low-Density-Lipoprotein (LDL). Liver and kidney function indicators, such as alanine aminotransferase (ALT), aspartate aminotransferase (AST), total bilirubin (TBIL), serum albumin (ALB), creatinine (Cr) and blood urea nitrogen (BUN) were detected using a fully automated biochemical analyser (Servicebio, Wuhan, China).

### Detection of related protein expression using laser scanning confocal microscopy

The gene sequence of eGFP was carried in an AAV8 vector. After the injection of Dil-Ox-LDL (Yiyuan Biotech, YB-0010) via the tail vein, cryosectioning of liver tissue was performed. After immunofluorescence staining, laser scanning confocal microscopy was used to detect the expression of LOX-1 and the phagocytosis of Ox-LDL by hepatocytes. The procedures were as follows. (1) The mice of LOX-1 and control groups were fasted for 8 h to obtain blood samples, followed by the injection of 30 μg/100 μl Dil-Ox-LDL (freshly prepared and protected from light during injection) into the tail vein. Thirty minutes later, mice were intraperitoneally anaesthetised and sacrificed. (2) For each mouse, the heart was exposed, and pre-cooled saline was used for cardiac perfusion. Aorta, heart, liver, spleen, lung and kidney specimens were collected. (3) For the cryosections, cryosectioning equipment was prechilled to − 20 °C, the thickness of each liver section was 10 μm, and 6 to 8 sections were collected from a liver sample. (4) After the sections were prepared, the nuclei were stained with 4′,6-diamidino-2-phenylindole (DAPI), and the sections were observed under a laser confocal microscope. The same parameters were used for each view. (5) Cryosections of aortic root tissue were slightly different, with a slice thickness of 6 μm, and cryosections of spleen, kidney, and lung tissue were generated using the same method as that for the liver sections.

### Western blot

The expression of LOX-1 (Abcam, ab203246), ABCG5 (Boster, PB0381), ABCG8 (Boster, A01482) and SR-BI (GeneTex, GTX113645) in liver tissues were detected using western blot. Liver tissues were homogenized in RIPA buffer (AntGene, ANT060) supplemented with protease and phosphatase inhibitors at 4 °C for 30 min. After centrifugation at 12,000×*g* for 15 min at 4 °C, the supernatant was collected and measured using the PierceTM BCA Protein Assay Kit according to the instructions. After the system was prepared, it was electrophoresed on a 10% polyacrylamide SDS gel, and then transferred to the membrane. The membrane was blocked with 5% skimmed milk powder at room temperature for 2 h, and then it was incubated in the indicated antibody at 4 °C overnight. The next day, incubate with horseradish peroxidase-conjugated secondary antibody for 2 h at room temperature, and then observe using ECL Kit. Chemiluminescence was detected by exposure to film and quantified using Image Lab.

### Quantitative real-time polymerase chain reaction (qRT-PCR) analysis

Total RNA was extracted from livers using Trizol reagent (Invitrogen, 15596026) according to the producer’s protocol. To obtain cDNA, we used a SYBR Premix Ex TaqTM Kit (Takara, RR420A) on a thermocycler (Bio-Rad, Hercules, CA, USA). The primer sequences were: GAPDH (forward) 5ʹ-ATGGGTGTGAACCACGAGA-3ʹ and (reverse) 5ʹ-CAGGGATGATGTTCTGGGCA-3ʹ; SR-BI (forward) 5ʹ-AGTTGGTGAGATCCTGTGGG-3ʹ and (reverse) 5ʹ-TCTTGCTGAGTCCGTTCCAT-3ʹ; ABCG8 (forward) 5ʹ-CTGGCACCCCTATCTACCTG-3ʹ and (reverse) 5ʹ-TTCTAGGAACAGGGCTGCAA-3ʹ; ABCG5 (forward) 5ʹ-AGAACAACACGCTAAAGGGC-3ʹ and (reverse) 5ʹ-GAAAATGACCGTGGCGATGA-3ʹ The mRNA expression levels of SR-BI,ABCG8 and ABCG5 were determined by comparing that of the GAPDH, which was chosen as internal controls.

### H&E staining

As previously described (Wang et al. 2021), H&E staining was conducted to assess cellular morphology in the tissue. Images were captured by a Nikon Eclipse microscope with the NIS Elements software.

### Oil Red O staining

The aorta perivascular adipose tissue and the surrounding connective tissue were carefully dissected under a stereomicroscope, and the portion from the aortic arch to the iliac artery bifurcation was completely preserved. After fixation with 4% paraformaldehyde, the aorta was stained with Oil Red O. After staining, digital images were taken with a microscope and stored. Plaque area was observed, calculated and analyzed using Image-Pro Plus 6.0 (The results are expressed as the percentage of the lesion area with lipid accumulation to the total gross area of the aorta). To further detect the lesion of atherosclerosis, Oil Red O staining were also used on serial sections (6 μm thick) of aortic root. Then digital images were acquired under low-magnification and high-magnification fields of an optical microscope (the view in which all of the tricuspid valve was visible was retained). Plaque area was observed under a microscope and calculated (results were expressed as the percentage of the lesion area with lipid accumulation to the total lumen area), and the results were statistically analyzed using Image-Pro Plus 6.0 software.

### Immunohistochemistry and immunofluorescence

To determine whether the liver had expressed LOX-1 (Proteintech, BC022295) successfully, we used immunohistochemistry and immunofluorescence staining to assessed it. The specific steps are as previously described (Sassi et al. 2017). For immunohistochemistry staining, serial sections of the liver were immunostained with antibody against LOX-1 (Proteintech, BC022295). And for immunofluorescence staining, serial sections were incubated with antibody LOX-1 (Proteintech, BC022295). To verify that hepatocytes can phagocytose Ox-LDL after expressing LOX-1, we performed immunofluorescence co-localization of the Dil-Ox-LDL and LOX-1. The specific steps are as described above. Serial sections were stained by using DAPI to observe nuclei. And the localization of LOX-1 in hepatocytes by using antibody against LOX-1 (Proteintech, BC022295). Since eGFP (green) and Dil-Ox-LDL (red) are self-fluorescent, the slices can be directly placed under a laser confocal microscope for the fluorescence analyses after staining LOX-1 (purple).

The extent of VCAM-1 (Servicebio, GB11336) and the migration of macrophages were determined by immunofluorescence staining. The specific steps are as described above. The migration of macrophages were observed by the co-localization of MCP-1 (Servicebio, GB11199) and CD68 (Servicebio, GB14043).

In addition, to verify whether the phagocytosis of Ox-LDL induce hepatocyte apoptosis, we detected the expression of TUNEL in liver by immunofluorescence. The specific steps are as described above.

### Dihydroethidium staining for reactive oxygen species (ROS) detection

To determine the levels of ROS, liver sections were incubated with Dihydroethidium (DHE) (D7008, Sigma-Aldrich, Saint Louis, Mo, USA) for 30 min at 37 °C. In the presence of ROS, DHE is oxidized to hydroxyethidium giving red fluorescence. Briefly, 5 mM DHE was applied to the liver sections and thereafter in situ fluorescence was analyzed using fluorescence microscopy. To quantify DHE staining, fluorescence intensity was analyzed in 4 fields/sections by Image-Pro Plus software.

### Statistical analysis

Quantitative data were collected from at least three independent replicates, and the measurement data are expressed as the mean ± standard deviation (SD). All data were statistically analyzed using SPSS 22.0 statistical software. As for the methods of statistical, the student’s t-test or one-way analysis of variance (ANOVA) was used when the data were normally distributed with homogeneity of variance. P < 0.05 was considered statistically significant.

## Results

### Adeno-associated virus with specific promoter TBG has good tissue specificity

**Fig. 1:**
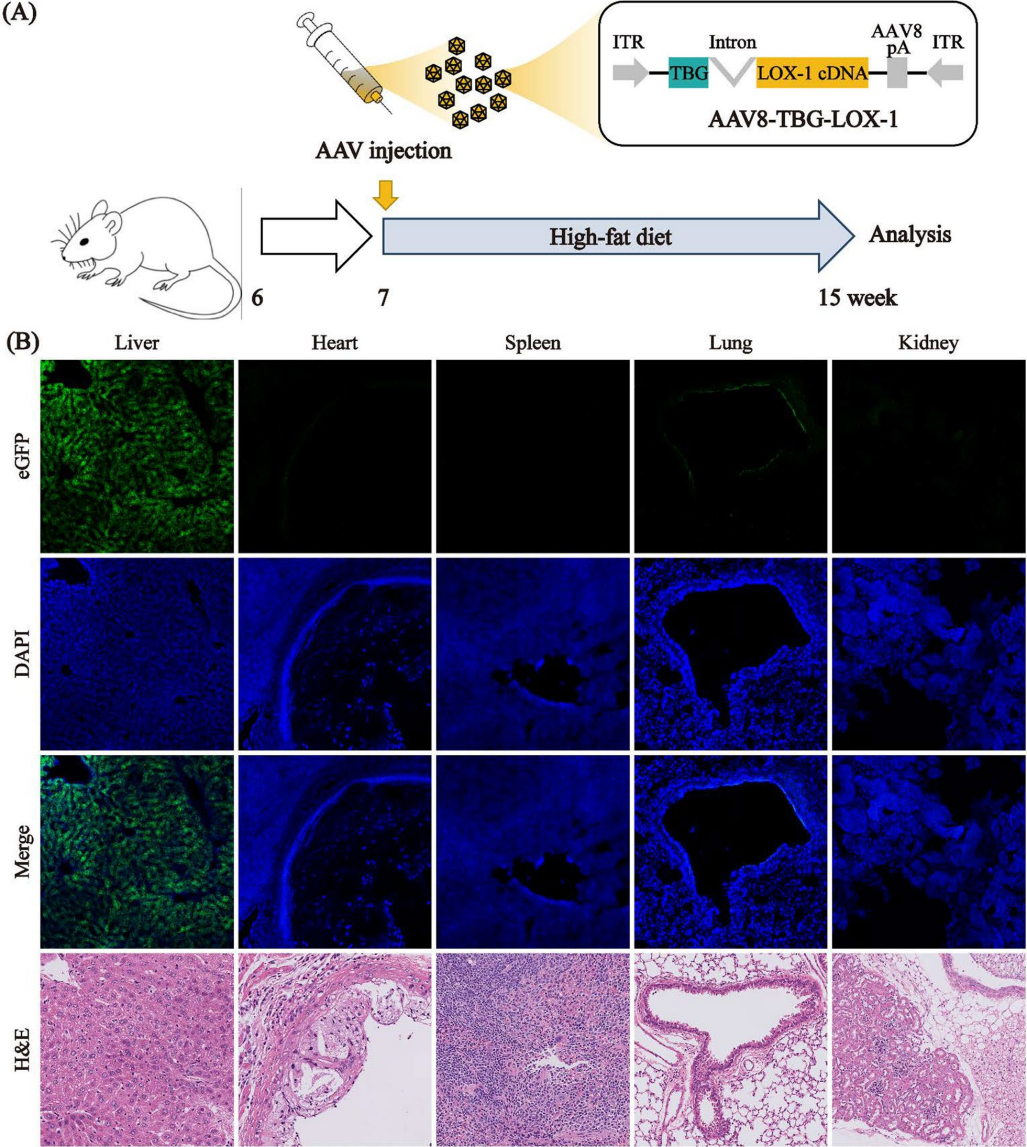
Construction and in vivo specific transfection of AAV8-TBG-eGFP. **A** Schematic showing the experimental strategy for high-fat diet feeding and analysis. **B** AAV8 carrying the TBG promoter can mediate the specific expression of target genes in the liver without affecting other organs in ApoE^−/−^ mice (laser scanning confocal microscopy, ×400)

To achieve liver-specific transfection, adeno-associated virus type 8 (AAV8) which has been proved to be highly efficient in liver transfection and liver-specific promoter TBG were selected to achieve liver-specific ectopically expressing LOX-1. Figure 1A shows the construction design for the AAV8-TBG-LOX-1 vector and a flow chart of the whole experiment. The AAV8-TBG-eGFP vector was injected through the tail vein to determine the specificity. Mice heart, liver, spleen, lung, kidney were collected, cryosectioned, and viewed under a laser scanning confocal microscope (Fig. 1B). To show the tissue and cell structure of the corresponding organs more clearly, we use H&E staining and fluorescent staining to correspond to each other (Fig. 1B). Under the same imaging parameters, abundant eGFP expression was observed in the liver of the mice, almost no eGFP was expressed in the heart, spleen and kidney, and a small amount of eGFP expression was observed in the lungs. These results indicated that the AAV8 vector carrying the liver-specific TBG promoter mediated the specific transfection of target genes in the liver, with very little impact on other organs and tissues.

### The stable expression of LOX-1 in the liver and LOX-1 phagocytosis of circulating Ox-LDL

**Fig. 2:**
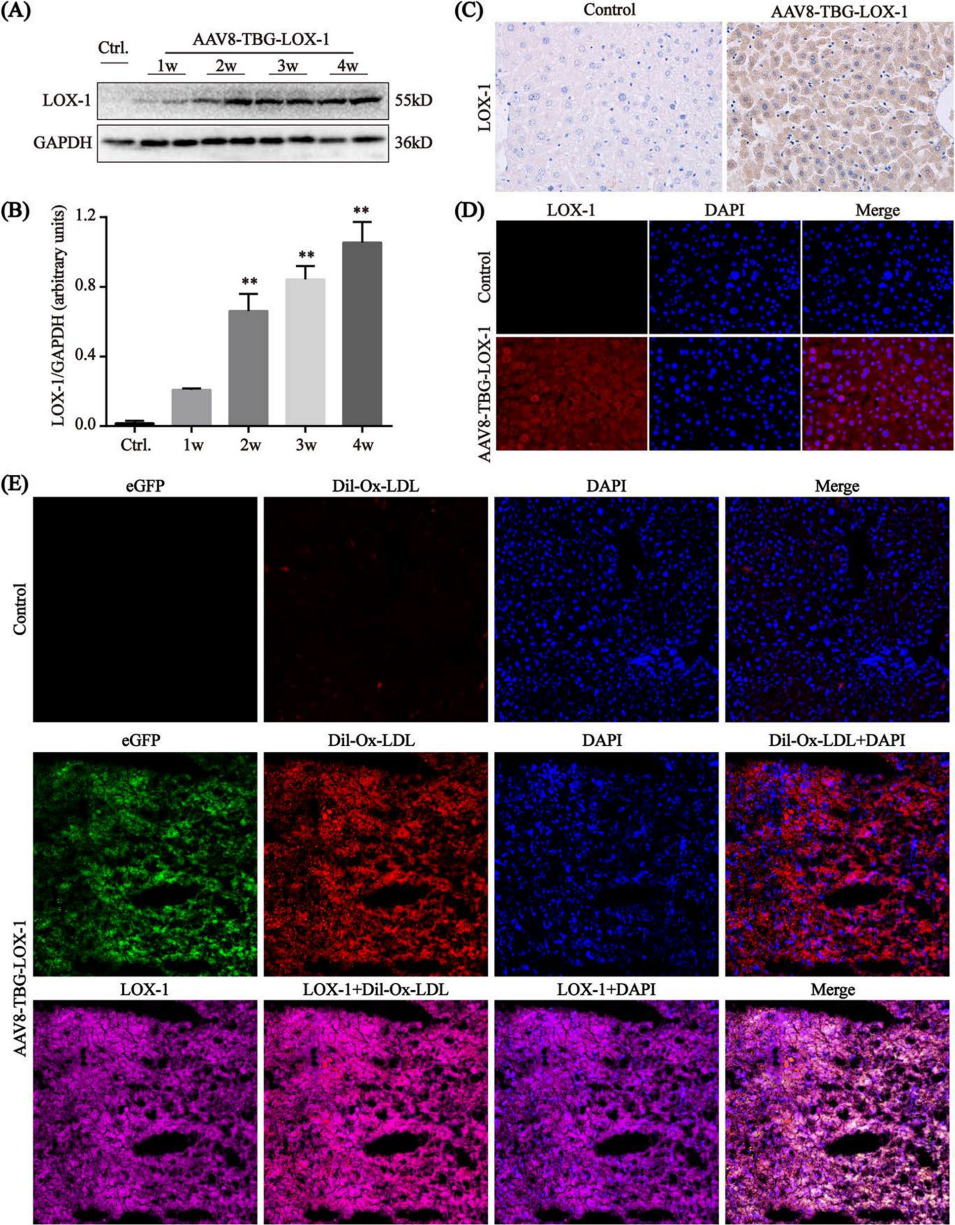
Functional expression of LOX-1 in the liver. **A**, **B** Relative protein expressions of LOX-1 in liver tissue of mice at different time points after transfection in LOX-1 group using western blot analyses. **P < 0.01 compared with the control group. **C** Detection of LOX-1 expression in liver tissue from mice in control and LOX-1 group (immunohistochemistry, n = 7, ×400). **D** Detection of LOX-1 expression in liver tissue from mice in control and LOX-1 group (immunofluorescence, n = 7, ×400). **E** Detection of LOX-1 expression in liver tissue and LOX-1-mediated Ox-LDL phagocytosis in hepatocytes (laser scanning confocal microscopy, n = 7, ×200)

The effects of transduction on the liver were assessed using western blot. The results (Fig. 2A, B) showed that compared with that in the control group, hepatic LOX-1 expression in the LOX-1 group was low at the 1st week and increased at the 2nd week, with stable expression observed at the 4th week. In order to see the localization of LOX-1 in hepatocytes more clearly after transfection, we used immunohistochemistry and immunofluorescence staining for detection (Fig. 2C, D). The results showed that LOX-1 was mainly expressed in the cell membrane and cytoplasm of hepatocytes after transfection, and most of the hepatocytes were successfully transfected. Under the laser confocal microscope (Fig. 2E), we observed that compared with the control group, the expression of eGFP (green fluorescence) in the liver of the LOX-1 group was obvious, indicating that the virus has been successfully transfected. Meanwhile, LOX-1 (purple fluorescence) was successfully expressed in the cytoplasm and cell membrane of hepatocytes in the virus group, and the results of co-localization with Dil-Ox-LDL (red fluorescence) showed that LOX-1 successfully engulfed Ox-LDL into the hepatocyte cytoplasm. From these results, we see that LOX-1 expressed in hepatocytes after transfection can phagocytose circulating Ox-LDL.

### Expression of LOX-1 in the liver can reduce circulating Ox-LDL without affecting blood lipid levels

**Fig. 3:**
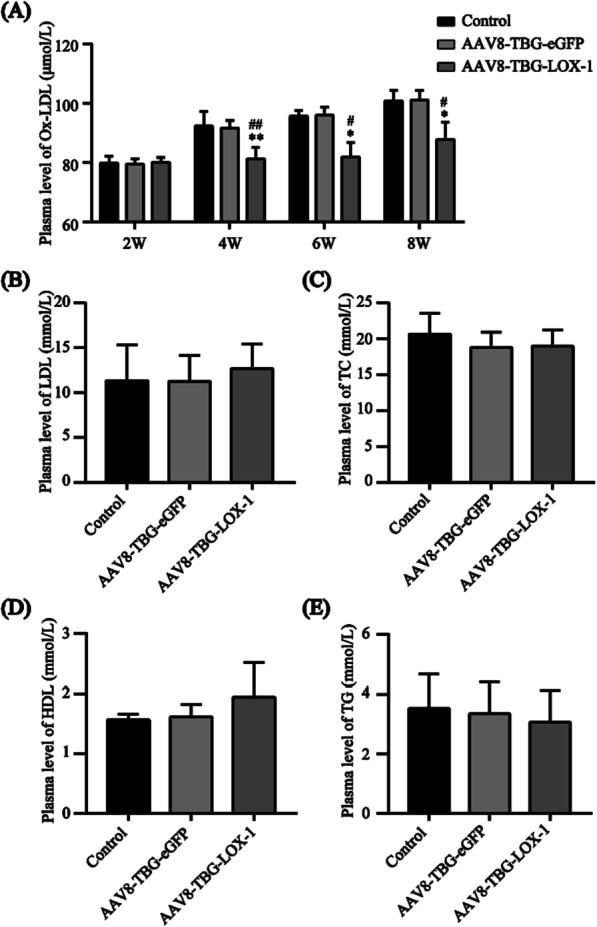
Ox-LDL and lipid levels in plasma. **A** Detection of Ox-LDL in circulating plasma (ELISA, n = 7). **B** Plasma LDL-C levels in mice 8 weeks after virus transduction (n = 7). **C** Plasma TC levels in mice 8 weeks after virus transfection (n = 7). **D** Plasma HDL-C levels in mice 8 weeks after virus transfection (n = 7). **E** Plasma TG levels in mice 8 weeks after virus transfection (n = 7). Values are expressed as mean ± SD (n = 7). *P < 0.05 and **P < 0.01 compared with the control group. ^#^P < 0.05 and ^##^P < 0.01 compared with the eGFP group

Plasma Ox-LDL was detected at the 2nd, 4th, 6th, and 8th weeks after virus transduction. As shown in Fig. 3A, after 8 weeks of a high-fat diet, plasma Ox-LDL in the control group and eGFP group rapidly increased within 4 weeks and slowly increased after 4 weeks and plasma Ox-LDL in the LOX-1 group was slightly higher than the initial level at the 2nd week. The results show that compared with those in the control and eGFP group, the plasma levels of TC, TG, and LDL-C in the LOX-1 group were lower, while the plasma level of HDL-C was higher, but none of the differences were statistically significant (Fig. 3B–E). These results suggested that hepatic LOX-1 expression can reduce Ox-LDL in plasma by recognizing, binding, and phagocytosing Ox-LDL without significantly affecting TC, TG, LDL and HDL levels in the plasma.

### Expression of LOX-1 in the liver does not cause significant side effects in vivo

**Fig. 4:**
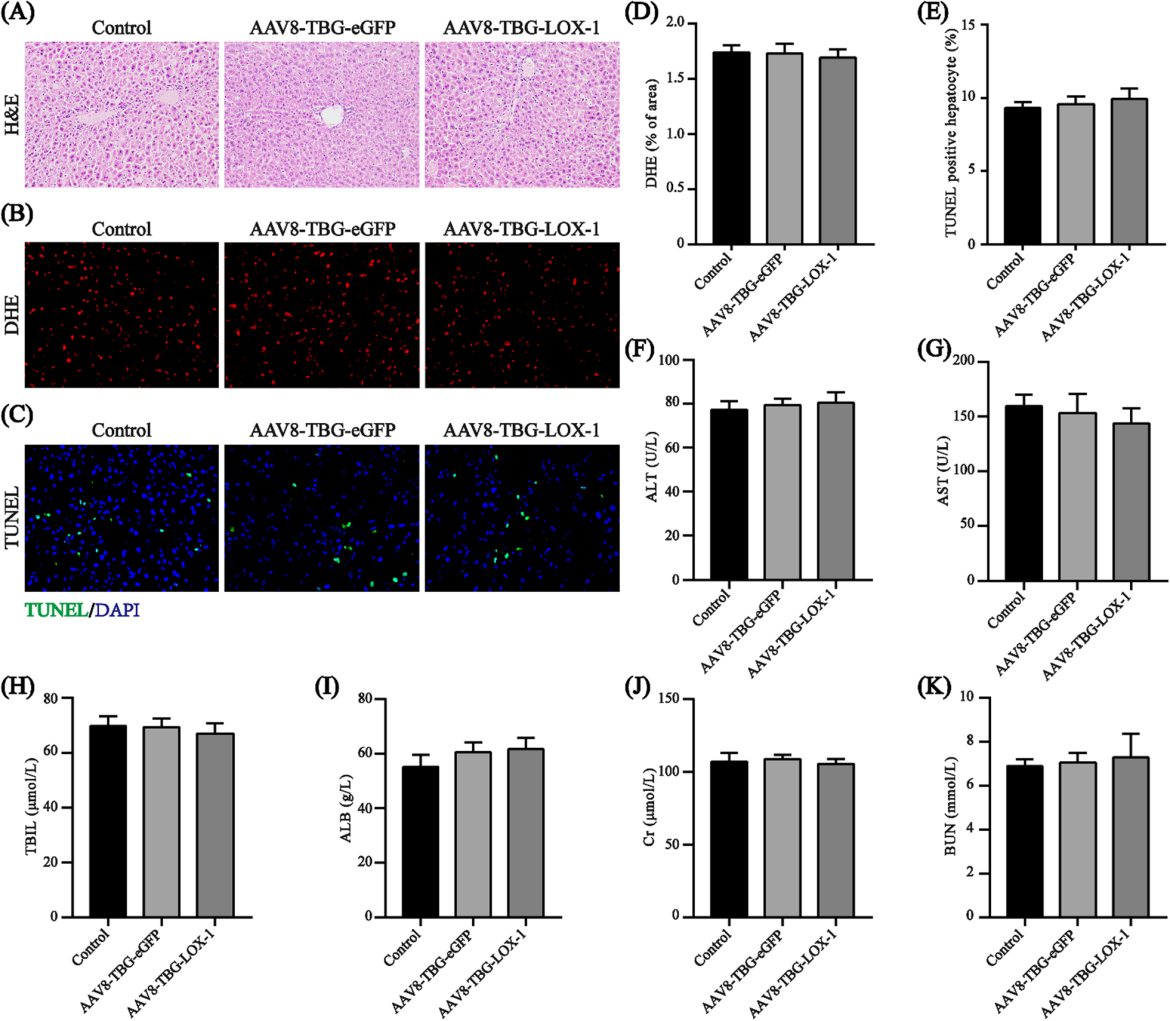
The injection of the virus dilution did not cause significant side effects in vivo. **A** H&E staining of liver sections 8 weeks after virus transfection (n = 7, ×200). **B**, **D** DHE staining of liver sections 8 weeks after virus transfection (n = 3, ×400) and quantification of DHE-positive areas in three study groups. **C**, **E** TUNEL immunofluorescent staining of liver sections 8 weeks after virus transfection (n = 3, ×400) and quantification of TUNEL-positive hepatocytes in three study groups. **F**–**K** Plasma ALT, AST, TBIL, ALB, Cr, BUN levels. Values are expressed as mean ± SD

Liver sections from mice in the control, eGFP and LOX-1 groups were stained with H&E to observe the effect of viral transfection on liver structure. As shown in Fig. 4A, 8 weeks after viral transfection, the morphology and structure of hepatocytes in all three groups did not change significantly. In addition, to further verify whether the phagocytosis of Ox-LDL can induce hepatocyte apoptosis or increase oxidative stress, we tested the corresponding indicators. From the results of DHE staining, we can see that, after 8 weeks virus transfection, the production of reactive oxygen specie (ROS) hadn’t increased in the LOX-1 group compared with the control and eGFP groups (Fig. 4B, D). And the result of TUNEL was also consistent with that of DHE staining (Fig. 4C, E). Changes in serum levels of key indicators of liver function (ALT, AST, TBIL and ALB) and renal function (Cr, BUN) in all three groups after 8 weeks virus transfection were assessed using a fully automated biochemical analyser to evaluate the safety of the virus (Fig. 4F–K). The data indicated that compared with those in the control and eGFP groups, liver function and renal function indicators in the LOX-1 group changed slightly, but no significant difference was identified. Therefore, according to the above results, it is reasonable to speculate that there will be no obviously side effects of virus transfection to make hepatocytes ectopically expressed LOX-1 receptor to phagocytose Ox-LDL from the circulation.

### Clearance of circulating Ox-LDL by ectopic expression of LOX-1 in the liver significantly inhibited atherosclerosis

**Fig. 5:**
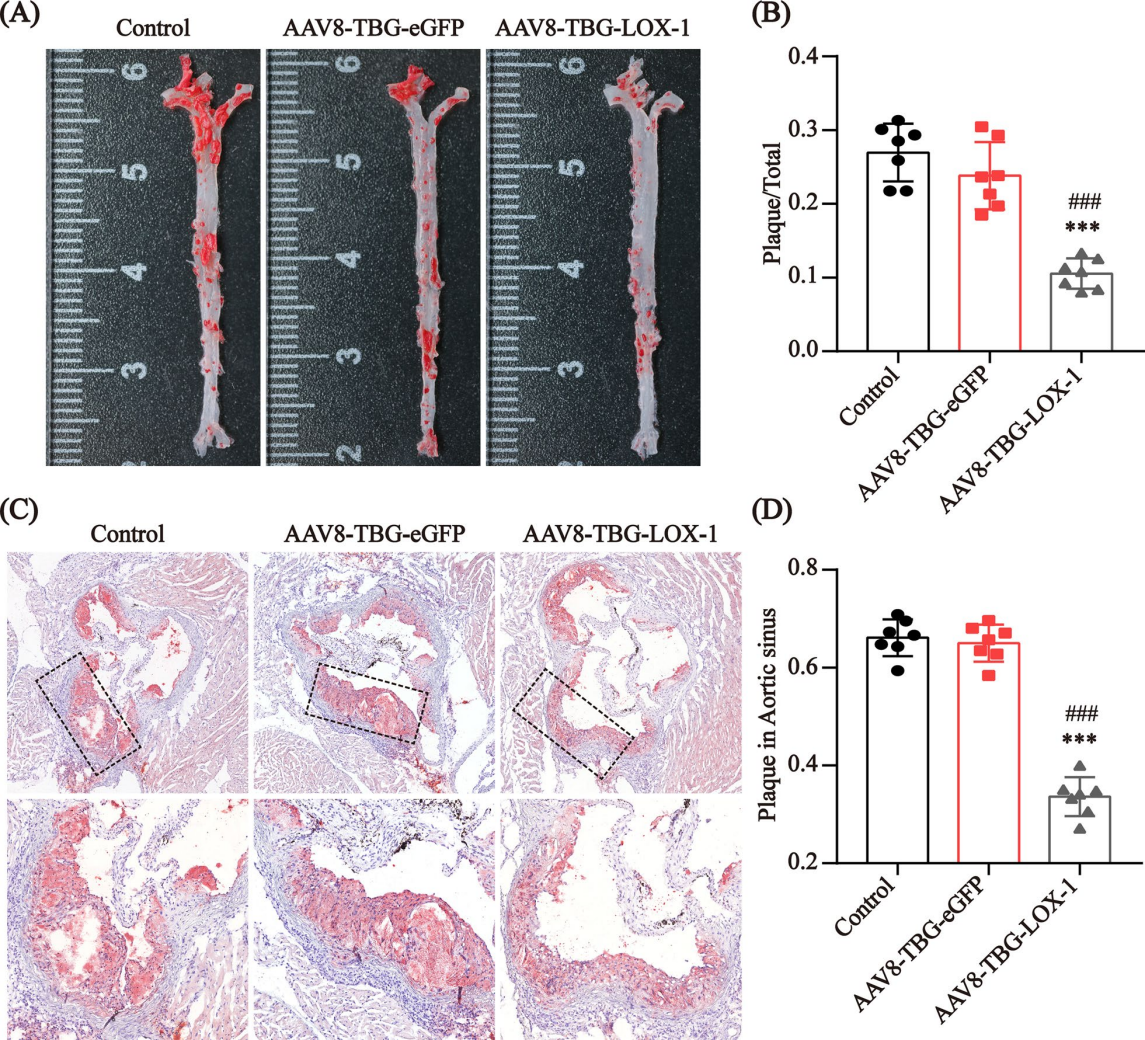
Effects of hepatic LOX-1 expression on atherosclerosis. **A**, **B** Representative images of aorta obtained from the study groups stained with Oil red O (**A**). The percentage of the plaque area in the aorta is presented among the indicated groups (n = 7) (**B**). **C**, **D** Representative images of aortic root sections obtained from the study groups stained with Oil red O (**C**). The percentage of the plaque area in the aortic root is presented among the indicated groups (n = 7). **D** Values are expressed as mean ± SD. ***P < 0.001 compared with the control group. ^###^P < 0.001 compared with the eGFP group

To determine the effect on atherosclerosis of clearance of circulating Ox-LDL, we observed the plaques in the aorta and aortic root sections using Oil red O staining. The percentages of aortic plaque area in mice in the control group, eGFP group and LOX-1 group were 26.9 ± 4.9%, 23.8 ± 4.5% and 10.5 ± 2.1%, respectively (Fig. 5A, C); the percentage of aortic plaque area in the LOX-1 group was much lower than that in the control and eGFP groups. For the aortic root (Fig. 5B, D), at the same magnification, the plaques in the control group were obvious, with cholesterol crystals present, and the plaques in the LOX-1 group were significantly smaller; the percentages of aortic root plaques in the mice in the control, eGFP and LOX-1 groups were 66.1 ± 3.8%, 65.0 ± 3.8% and 33.6 ± 3.9%, respectively. These results suggested that the ectopic expression of LOX-1 in the liver to clear circulating Ox-LDL indeed inhibited the formation of atherosclerosis.

### Clearance of circulating Ox-LDL by ectopic expression of LOX-1 in the liver significantly reduce migration of macrophages in plaques and VCAM-1 expression in vascular endothelium

**Fig. 6:**
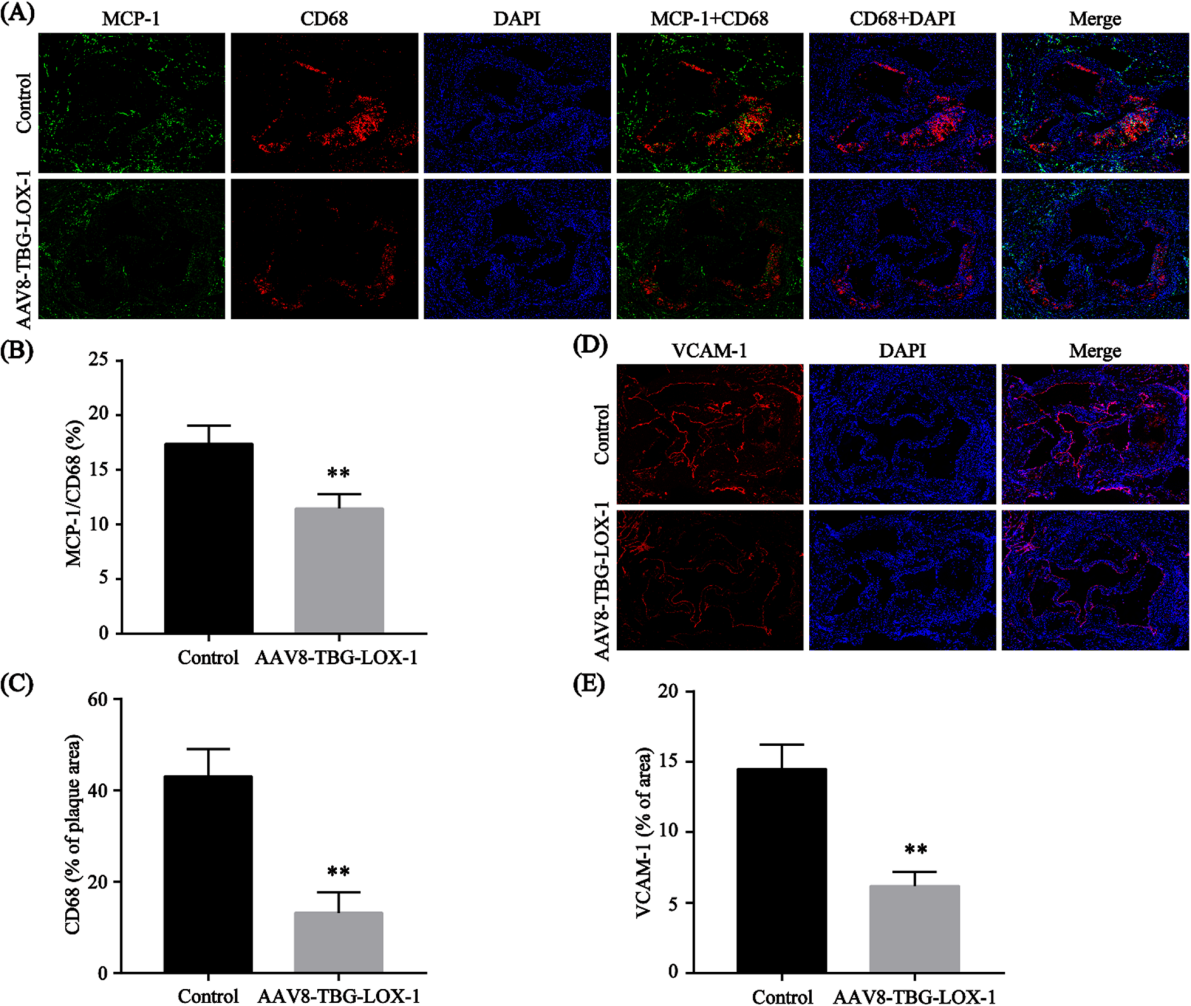
Clearance of circulating Ox-LDL by ectopic expression of LOX-1 in the liver significantly reduce migration of macrophages in plaques and VCAM-1 expression in vascular endothelium. **A**–**C** Representative images of the co-localization of MCP-1 and CD68 in the plaque obtained from the control and LOX-1 groups by using immunofluorescence (**A**). DAPI were used to stain nuclei (blue). The percentage of MCP-1/CD68 is presented among the indicated groups (**B**). The percentage of the plaque area that was occupied by CD68 is presented among the indicated groups (**C**). **D**, **E** Representative images of the plaque in the control and LOX-1 groups stained with VCAM-1 (**D**). DAPI were used to stain nuclei (blue). The percentage of the aortic root area that was occupied by VCAM-1 is presented among the indicated groups (**E**). Values are expressed as mean ± SD (n = 7). **P < 0.01 compared with the control group

To further determine the effect of clearing the circulating Ox-LDL, we also detected the migration of macrophages and the expression of VCAM-1 in vascular endothelium. The results suggested that compared with the control group (43.07% ± 5.962%), the content of macrophages in the plaque of the LOX-1 group (7.77% ± 5.361%) was significantly reduced (Fig. 6A, C). And according to the co-localization of monocyte chemoattractant protein-1 (MCP-1) and CD68, we can see that the migration of macrophages was also inhibited in the LOX-1 group (11.46% ± 1.32% vs. 17.38% ± 1.677%) (Fig. 6A, B). Consistent with this, the expression of VCAM-1 in the LOX-1 group (6.17% ± 1.042% vs. 14.49% ± 1.759%) was also greatly reduced (Fig. 6D, E). Based on these results, we reasonably speculated that elimination of Ox-LDL by hepatocytes can reduce the activation of vascular endothelial inflammation and subsequent subendothelial migration of macrophages, which could help to reduce intravascular inflammation and foam cell formation.

### Endocytosis of Ox-LDL by hepatocytes upregulates the expression of ABCG5 and ABCG8 in the cholesterol biliary excretion pathway

**Fig. 7:**
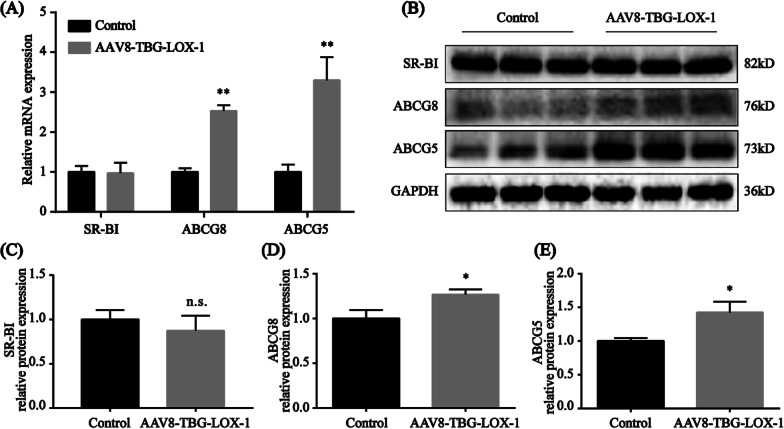
Quantitative real-time PCR and Western blot analysis of SR-BI, ABCG5, and ABCG8. **A** Quantitative real-time PCR analysis of the mRNA levels of SR-BI, ABCG8 and ABCG5 in liver of the mice in the control and LOX-1 groups. The expression of mRNA was normalized to that of GAPDH. **B**-**E** Western blot analysis of the hepatic lipid metabolism core pathway-related proteins SR-BI, ABCG5, and ABCG8 8 weeks after virus transduction. Values are expressed as mean ± SD. **P < 0.01 compared with the control group

To explore the possible mechanisms of the degradation of Ox-LDL in the hepatocytes, we investigated two possible pathways, biliary cholesterol excretion and reverse cholesterol transport. Based on the previous studies, we know that the ABCG5/G8 heterodimer is the primary neutral sterol transporter in hepatobiliary and transintestinal cholesterol excretion (Zein et al. 2019). And SR-BI binds HDL with high affinity, is expressed primarily in liver and nonplacental steroidogenic tissues, and mediates selective cholesterol uptake by a mechanism distinct from the classic LDL receptor pathway (Acton et al. 1996). So we examined the expression of representative molecules ABCG5, ABCG8 and SR-BI to observe the change of these two pathways. In our study, we found that, compared with the control group, the mRNA level of SR-BI almost remained unchanged in the LOX-1 group. However, the mRNA level of ABCG8 and ABCG5 were obviously higher in the LOX-1 group (Fig. 7A). The results of western blot indicated that compared with those in the control group, the expression level of SR-BI, the protein in the reverse cholesterol transport pathway, was not significantly different and the expression levels of ABCG5 and ABCG8, markers of the biliary cholesterol excretion pathway, were also increased in the LOX-1 group (Fig. 7B–E). Accordingly, we reasonably speculated that after the uptake of Ox-LDL by hepatocytes via LOX-1, Ox-LDL was degraded into amino acids and free cholesterol in lysosomes, and cholesterol was secreted into the intestinal lumen in the form of bile acids with the help of cholesterol transporters ABCG5/8.

## Discussion

The development and progression of atherosclerosis is closely related to the subendothelial deposition of lipids and chronic inflammation in the vascular wall (Libby et al. 2002). Despite tremendous achievements in the inhibition of atherosclerosis through modern medical drug treatment and lifestyle interventions, atherosclerosis still has the highest morbidity among diseases and is the leading cause of mortality in the world (Timmis et al. 2018). A more in-depth study of its pathophysiological mechanisms and the search for crucial intervention pathways and targets are necessary and urgent.

Ox-LDL is an important risk factor for atherosclerosis and is present in both atherosclerotic plaques and circulating plasma (Tsimikas and Witztum 2001). Ox-LDL phagocytosis and pathological processing through pattern recognition receptors on the vascular wall is the core mechanism of lipid-driven inflammation in the vascular wall. Ox-LDL activates mitogen-activated protein kinases (MAPKs) after endocytosis via LOX-1 in vascular endothelial cells, thereby increasing the expression of chemokine monocyte chemotactic protein 1 (MCP-1) and endothelial adhesion molecules. In addition, Ox-LDL promotes the subendothelial migration and differentiation of monocytes into macrophages (Kattoor et al. 2019; Pirillo et al. 2013). Ox-LDL downregulates the expression of the antiapoptotic protein B-cell lymphoma 2 (Bcl-2) through LOX-1 and activates cysteine-aspartic acid proteases (caspase-3 and caspase-9) in the apoptotic signalling pathway to promote endothelial cell apoptosis (Chen et al. 2004). Ox-LDL binds to LOX-1 and induces an increase in ROS expression and a reduction in carbon monoxide (NO) synthesis via the reductive coenzymes (NADPH) on the cell membrane, resulting in endothelial dysfunction (Ryoo et al. 2011). Therefore, the targeted clearance of Ox-LDL is a possible strategy to reduce inflammatory responses driven by lipid deposition in the vascular wall and inhibit atherosclerosis.

**Fig. 8:**
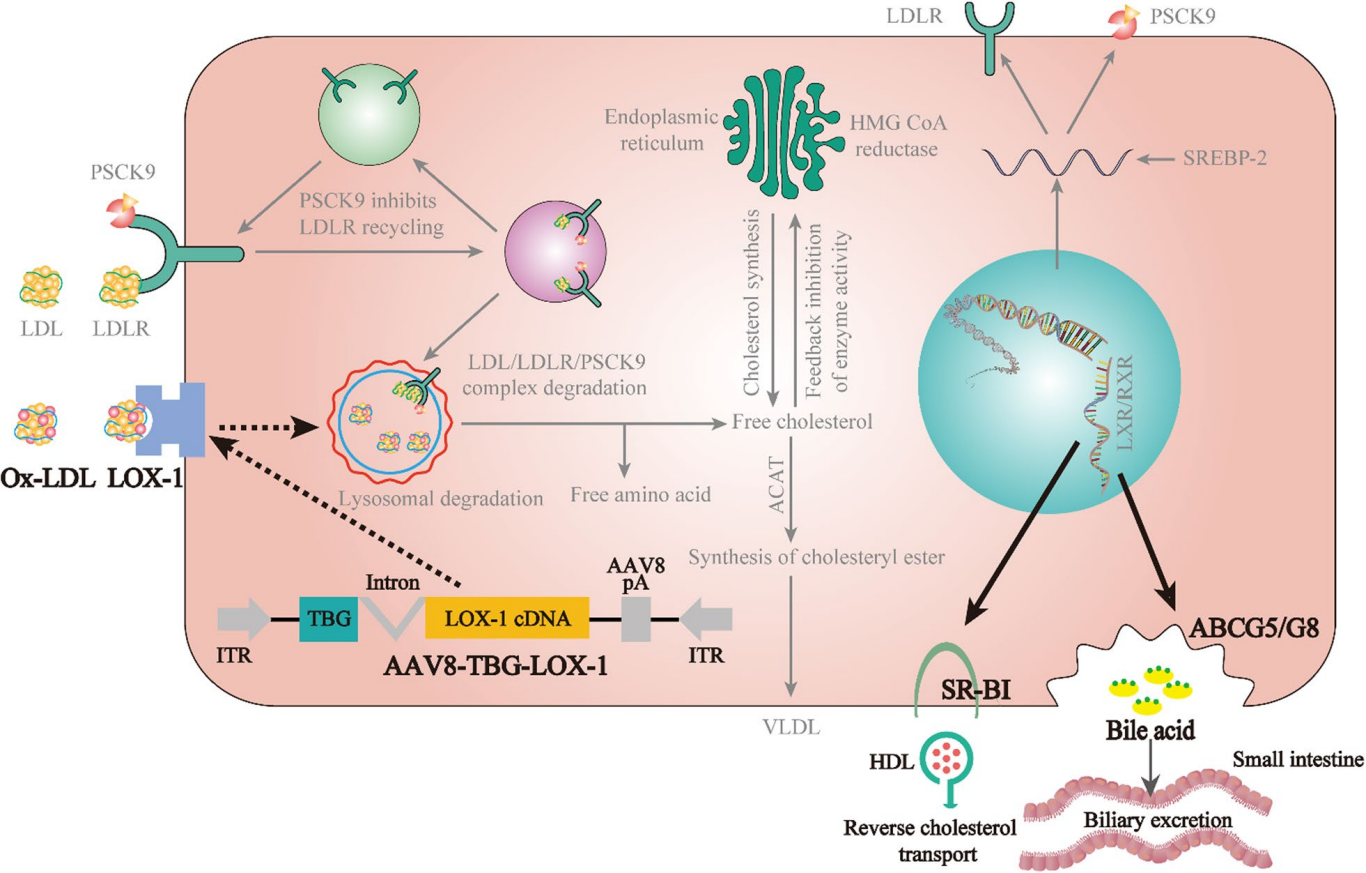
Schematic diagram of the Ox-LDL uptake and lipid metabolism pathways of the hepatocytes. AAV8-TBG-LOX-1 induces hepatocyte-specific expression of LOX-1, and hepatocytes endocytose LDL and Ox-LDL via LDLR and LOX-1; LDL and Ox-LDL are then degraded into amino acids and free cholesterol by lysosomes. Free cholesterol can induce an increase in biliary cholesterol excretion in hepatocytes; furthermore, it can reduce cholesterol synthesis through feedback inhibition of hydroxymethylglutaryl coenzyme A (HMG-CoA) reductase. Hepatocytes can maintain stable lipid metabolism through other cholesterol metabolism pathways, such as the reverse cholesterol transport pathway. Note: The entire square region is a hepatocyte, and the nucleus (green sphere) is the centre of metabolic regulation. The nuclear transcription factor LXR/RXR located in the nucleus regulates the expression of the cholesterol transporters ABCG5 and ABCG8, which secrete cholesterol into the lumen of the small intestine (the lower right area in the figure) as bile acids. SREBP-2 (RNA helix-like icon in the upper right of the figure) transcribed into the cytoplasm through the nucleus regulates the dynamic equilibrium between LDLR and PCSK9. LDLR (green arc-shaped transmembrane receptor in the figure), located on the cell membrane surface, is responsible for the uptake and metabolism of circulating LDL (near the cell membrane and cytoplasmic molecules and organelles in the upper left of the figure), and the ability of LDLR to recycle to the cell membrane after binding LDL and internalizing into hepatocytes is closely related to the regulation of PCSK9 (the red icon in the upper right of the figure). HDL-C is taken up through SR-BI (light green receptor with corresponding green spherical HDL icon in the lower right of the figure) on the surface of hepatocytes. In the endoplasmic reticulum (green reticular membrane icon in the cytoplasm in the middle-upper portion of the figure), HMG-CoA reductase synthesizes cholesterol, of which some is packaged into VLDL and secreted into the blood and some is stored after ACAT esterification

The liver is the core organ of lipid metabolism in the body and has well-developed lipid metabolism and regulatory pathways (Li et al. 2021; Ren et al. 2018). In particular, the liver has cholesterol degradation capacity and a mechanism to inhibit cholesterol synthesis. Furthermore, the liver also excretes biliary cholesterol, accounting for half of the synthetic cholesterol intake by the liver. Therefore, the specific ectopic expression of LOX-1 on the membrane of hepatocytes through AAV8-TBG is a unique way to achieve Ox-LDL degradation in the liver. In this study, based on the current research frontier and preliminary studies in our laboratory, we used an AAV8 vector carrying the liver-specific promoter TBG to mediate LOX-1 gene transfection in the liver of ApoE^−/−^ mice to ectopically express LOX-1, an Ox-LDL-specific receptor, in the liver. Circulating Ox-LDL can be persistently cleared via the robust lipid processing capacity of the liver, and rigorous experimental studies were conducted to investigate the effect of circulating Ox-LDL clearance on atherosclerosis. We found that persistent liver-specific ectopic expression of LOX-1 could not only recognize, bind to, and phagocytose circulating Ox-LDL but also inhibit atherosclerosis progression by upregulating the expression of the cholesterol transporters ABCG5 and ABCG8 to excrete cholesterol into the intestinal lumen as bile acids (Patel et al. 2018), thereby enabling Ox-LDL degradation and avoiding Ox-LDL deposition in the vascular wall and lipid-driven inflammatory responses. Of course, since endothelial cells will induce a series of damage after phagocytosis of Ox-LDL, we are also concerned about the state of hepatocytes after phagocytosis of Ox-LDL. However, according to our results, we found that 8 weeks after virus transfection, the level of oxidative stress and the occurrence of apoptosis in the liver of the LOX-1 group were not different from those of the control group. Therefore, we believe that the use of AAV8 to specifically transfect the liver to physiologically clear the circulating Ox-LDL did not cause significant damage to the liver. The aim of this study was to explore a safe and reliable pathway to eliminate Ox-LDL, via the physiological clearance of circulating Ox-LDL, for anti-atherosclerosis studies and to elucidate the possible mechanism of action. Figure 8 better shows our experimental assumptions. However, our study has some limitations. In subsequent experiments, we could use cyclization recombinase (Cre) mice to specifically knock down LOX-1 in the vascular endothelium for a more sophisticated exploration. In addition, the inflammatory responses within the body after the degradation of circulating Ox-LDL by the liver could be studied in more detail.

In summary, our research basis is the clearance of Ox-LDL, the pathogenic core of atherosclerosis. The ectopic expression of LOX-1 in the liver was achieved through gene therapy, resulting in plasma Ox-LDL clearance. The traditional Ox-LDL phagocytosis by pattern recognition receptors on endothelial cells in the vascular wall, leading to a lipid-driven inflammatory response, was modified to Ox-LDL metabolic degradation by liver-specific receptors, leading to biliary cholesterol excretion to the intestinal lumen. This will provide new targets and new breakthroughs for the investigation of atherosclerosis and provide new ideas for the clinical treatment of atherosclerosis.

## Conclusion

All in all, we propose a novel therapeutic direction for atherosclerosis, the physiological removal of Ox-LDL. Here in this study, we used the principle of gene editing to make the LOX-1 ectopically express in hepatocytes, and with the strong lipid metabolism ability of hepatocytes to clear Ox-LDL in the circulation, ultimately alleviating the progression of atherosclerosis.

## Data Availability

The data presented in this study are available on request from the corresponding author on reasonable request.

## Abbreviations

LOX-1: Lectin-like oxidized low density lipoprotein receptor 1
Ox-LDL: Oxidized Low-Density-Lipoprotein
ApoE: Apolipoprotein E-deficient
AAV8: Adeno-associated virus type 8
TBG: Thyroxine binding globulin
H&E: Hematoxylin–eosin
Dil-Ox-LDL: Dil-labeled Ox-LDL
VCAM-1: Vascular Cell Adhesion Molecule-1
qRT-PCR: Quantitative real-time PCR
ABCG5/8: ATP-binding cassette G5 and G8
LDL: Low-density lipoprotein
eGFP: Enhanced green fluorescent protein
PBS: Phosphate-buffered saline
TC: Total cholesterol
TG: Triglyceride
HDL: High-density lipoprotein
ALT: Alanine aminotransferase
AST: Aspartate aminotransferase
TBIL: Total bilirubin
ALB: Serum albumin
Cr: Creatinine
BUN: Blood urea nitrogen
MCP-1: Monocyte chemotactic protein-1
SR-BI: Scavenger receptor class B type I
ROS: Reactive oxygen species
DHE: Dihydroethidium
